# Topographic Independent Component Analysis reveals random scrambling of orientation in visual space

**DOI:** 10.1371/journal.pone.0178345

**Published:** 2017-06-22

**Authors:** Marina Martinez-Garcia, Luis M. Martinez, Jesús Malo

**Affiliations:** 1 Instituto de Neurociencias, CSIC, Alicante, Spain; 2 Image Processing Lab., Universitat de València, València, Spain; Tokai University, JAPAN

## Abstract

Neurons at primary visual cortex (V1) in humans and other species are edge filters organized in *orientation maps*. In these maps, neurons with similar orientation preference are clustered together in iso-orientation domains. These maps have two fundamental properties: (1) *retinotopy*, i.e. correspondence between displacements at the image space and displacements at the cortical surface, and (2) a trade-off between good *coverage* of the visual field with all orientations and *continuity* of iso-orientation domains in the cortical space. There is an active debate on the origin of these locally continuous maps. While most of the existing descriptions take purely geometric/mechanistic approaches which disregard the network function, a clear exception to this trend in the literature is the original approach of Hyvärinen and Hoyer based on infomax and Topographic Independent Component Analysis (TICA). Although TICA successfully addresses a number of other properties of V1 simple and complex cells, in this work we question the validity of the orientation maps obtained from TICA. We argue that the maps predicted by TICA can be analyzed in the retinal space, and when doing so, it is apparent that they lack the required *continuity* and *retinotopy*. Here we show that in the orientation maps reported in the TICA literature it is easy to find examples of violation of the continuity between similarly tuned mechanisms in the retinal space, which suggest a random scrambling incompatible with the maps in primates. The new experiments in the retinal space presented here confirm this guess: TICA basis vectors actually follow a random salt-and-pepper organization back in the image space. Therefore, the interesting clusters found in the TICA topology cannot be interpreted as the actual cortical orientation maps found in cats, primates or humans. In conclusion, Topographic ICA does not reproduce cortical orientation maps.

## 1 Introduction

### Retinotopy, continuity and coverage in V1 orientation maps

Neurons at the primary visual cortex in mammals are localized edge filters, i.e. their receptive fields are tuned to certain orientation at certain retinal location [1, 2]. The large scale organization of the V1 surface is *retinotopic*, i.e. the retinal space is mapped onto the cortical space so that neighbor cells in the V1 surface are tuned to close regions in the visual field [3–6]. At a finer scale, the organization is retinotopic as well [7–11]: spatial displacements in V1 (in *μ*m) correspond to spatial displacements in the visual field (in *degrees*). *Retinotopy* refers to the correspondence between the location of the receptive field at the retinal space and the location of the neuron at the cortex.

For some species like cats, primates and humans, on top of retinotopy, V1 cells have a quite special arrangement: the so called *orientation maps*. In these maps, neurons with similar orientation preference are clustered together in the so called iso-orientation domains.

On the one hand, these iso-orientation regions are locally *continuous*, i.e. neighbor cells share the same orientation preference except in the boundaries of the region (the fractures and pinwheels). There is certain level of noise (or receptive field scatter) in this continuous distribution, as reported in [12]. However, high-resolution imaging shows that this noise does not prevent the observation of large continuous regions of neurons clearly segregated by orientation tuning in certain species [10], versus others that follow a discontinuous random arrangement [13]. On the other hand, the iso-orientation domains are interleaved for a good *coverage* of the visual field, i.e. the domains are juxtaposed so that continuous patches of similarly tuned neurons can be found all over the visual field [8]. Note that *continuity* of the domains and good *coverage* of the visual field are opposed properties: strict continuity would imply large regions tuned to a specific orientation and hence a poor coverage of the field by neurons tuned to other orientations, while perfect coverage with all orientations is only possible with infinitesimally small (or discontinuous) regions.

In order to describe the functional maps in the mammalian brain, Wilson and Bednar [14] use the terms *topographic maps* and *topological maps*. While *topographic maps* refer to the spatial layout of sensory surfaces according to location (like the skin or the retina), *topological maps* refer to the spatial organization (or grouping) according to features of the sensory input more abstract than location. In the case of animals with structured orientation maps, these are *topological* because of the local continuity of iso-orientation domains, and they are also *topographic* because the *retinotopy* implies that distances in the image space have a well-defined relationship with distances across the cortical surface [14].

**Fig 1:**
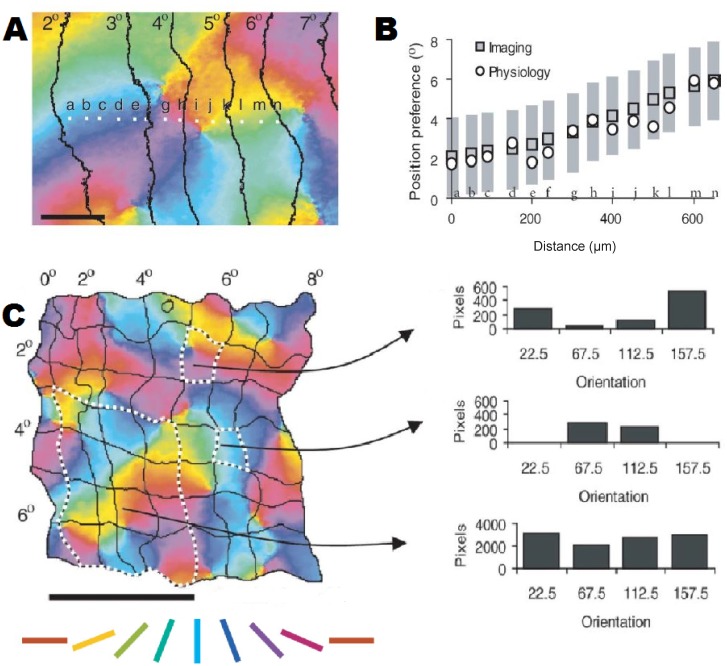
Retinotopy, continuity and coverage in V1 orientation maps (reprinted from [8] with permission of Nature Publishing Group). Subfigures A and C correspond to illustrative V1 surface regions in animals with orientation maps (in this case, tree shrews). Colored neighborhoods imply common orientation tuning properties of the neurons: the iso-orientation domains. The color code corresponding to subfigures A and C is below subfigure C. Black scale bars, 200 *μ*m (A), 1mm (C). Black curves in A represent iso-azimuth contours: the cortical location of the maximum response when stimulating with lines at linearly spaced visual angles in the retinal space. Similarly, the black grid in C represents the cortical location of an iso-azimuth/iso-elevation grid in the retinal space. The orientation domains and the iso-azimuth/elevation grids in A and C were obtained using optical imaging. Moreover, multi-unit electrophysiological recording was used to measure the preferred position (central location of the receptive field in the retinal space) at the cortical locations highlighted with letters in subfigure A. Displacements along the cortex imply equivalent displacements (of the receptive fields) along the visual space given the linear relation in subfigure B. Finally, histograms in subfigure C represent the number of neurons tuned to specific orientations in small and large regions of the cortex (or equivalently in small or large visual regions). Continuity of the domains implies peaks in the histograms of small regions, while good coverage of the visual field by different domains implies more uniform histograms when larger visual/cortical regions are considered.

Fig 1 replotted from [8] summarizes the basic experimental properties of orientation maps in the visual cortex of many mammals including cats, primates and humans: (1) *retinotopy*, i.e. correspondence between displacements at the image space and displacements at the cortical surface, and (2) a trade-off between *continuity* of each iso-orientation domain, and good *coverage* of the visual field with the different iso-orientation domains.

The grid and retinotopy illustrated in Fig 1 lead to the following key property: *maps measured at V1 have a retinal counterpart*. Note that the receptive fields of a set of neurons located in a grid in V1, arranged in orientation domains, induce an equivalent grid and equivalent orientation domains in the visual space. This is because the retina-V1 transform is smooth: the slightly distorted black grid at the cortex in subfigure C corresponds to a regular grid back in the retinal space. Therefore, one can imagine equivalent orientation domains, with equivalent continuity and coverage, formed by the centers of the receptive fields in the spatial domain. As a consequence, the models should not only reproduce the arrangement in the cortex (i.e. outputs of the processing units organized in orientation maps), but also in the retinal space (i.e. the centers of the receptive fields of the predicted units have to be arranged in locally continuous maps according to their orientation tuning).

### A functional approach to orientation maps

There is an active debate on the origin of the local continuity of the orientation maps: *where does it come from?*, *what is its purpose?* [14, 15]. Note that these questions are different in nature: while the first is mechanistic, the second is a functional question.

Most of the literature follows purely mechanistic approaches, in fact there are interesting competing alternatives to describe how the orientation domains arise: (i) as a result of Moire patterns from the structure of the retinal ganglion cells [16, 17] (questioned in [18, 19]), (ii) as a result of self-organization achieved by Hebbian learning and training with natural images [20–22], and (iii) as a result of the local connections leading to global regularities, earlier formulations in [23, 24] and more recent versions based on reaction diffusion in [25] (revisited in [26, 27]). All these works build a mechanism or model which under certain constraints (wiring, connectivity, continuity, coverage, etc.) gives rise to the maps, but these models are not related to the information processing function carried out by the network, nor clarify the functional advantage of having iso-orientation domains [14].

A remarkable exception is the work of Hyvärinen and Hoyer [28] which is based on a functional argument: the network should maximize the transmitted information. Information maximization (or infomax) implies that the responses of the different neurons to natural images should be as statistically independent as possible [29, 30]. Under this functional constraint, the linear Independent Component Analysis (ICA) is able to explain the shape of the receptive fields [31]. However, the linear nature of ICA prevents the complete achievement of the infomax goal, and residual statistical dependence does remain after linear ICA in natural images [32, 33].

Hyvärinen and Hoyer extended linear ICA by taking into account the residual statistical dependence after linear ICA. This is the core of the so called Topographic-ICA (TICA) [34]. In TICA, the correlations between the energies of first-stage linear filters are used to build a second-stage 2-dimensional cyclic topology (or grid) in which the processing units with stronger relations are closer in the grid. As a result, clusters of units tuned to similar features naturally emerge in this topology.

When applied to natural images, TICA certainly accounts for the basic features of both simple and complex cells in V1 [28], e.g. first-stage units have localized and oriented edge receptive fields (as the simple cells), and second-stage units have broader phase and location tuning (as complex cells). The authors also report the emergence of *orientation maps* derived from TICA, displaying iso-orientation domains and pinwheels, which appear again in [35, 36]. However, the orientation maps resulted in [28, 35, 36] may not be appropriate to explain orientation maps in V1 since they may lack the appropriate continuity and retinotopy.

The continuous orientation maps reported in [28, 35, 36] are found in the topology emerging from the algorithm (assumed to be the cortical space), but this topology can not be automatically transferred into the retinal space. Since the location in the TICA topology may not be automatically interpreted as retinal location, the TICA maps may not be equivalent to the biological maps (which are genuinely retinotopic and continuous). Hyvarinen & Hoyer do recognize that the retinotopy of the TICA topology is only local [28], but the implications of this fact were not discussed. No additional analysis has been done to confirm or refute the biological plausibility of the clusters emerging in the topology. In this work we elaborate on this open question: we argue that a biological interpretation of the predicted clusters would require a smoother retinotopic relation between the retinal space and the TICA topology, because, as opposed to the animals with map patterns, the TICA orientation maps does not have an appropriate counterpart in the image domain with locally continuous structure.

Specifically, in the examples below we show that the topology reported in [28] (and sequels [35, 36]) violates the continuity of the orientation maps in such a way that, given two close units (artificially predicted neurons) in the TICA topology, it is easy to find another unit tuned to the same spatial position and an orthogonal orientation. Here, spatial position tuning means the center of the receptive field in the retinal space. These examples suggest non-continuous, or randomly scrambled, orientation domains when projecting them back to the visual space. In order to check this intuition, in this work we conduct a new analysis of the units emerging from TICA: we compute the spatial distribution of oriented units in the retinal space. The invariable result (over a wide set of training conditions) is a random salt-and-pepper organization, thus confirming the intuition obtained from the particular examples of continuity violation detected in the results of [28, 35, 36].

This salt-and-pepper organization of the TICA oriented filters in the retinal space implies that the locally continuous TICA topology does not have a locally continuous counterpart in the image space, and hence, it is not biologically plausible.

### Examples of continuity and retinotopyviolation in the TICA literature

**Fig 2:**
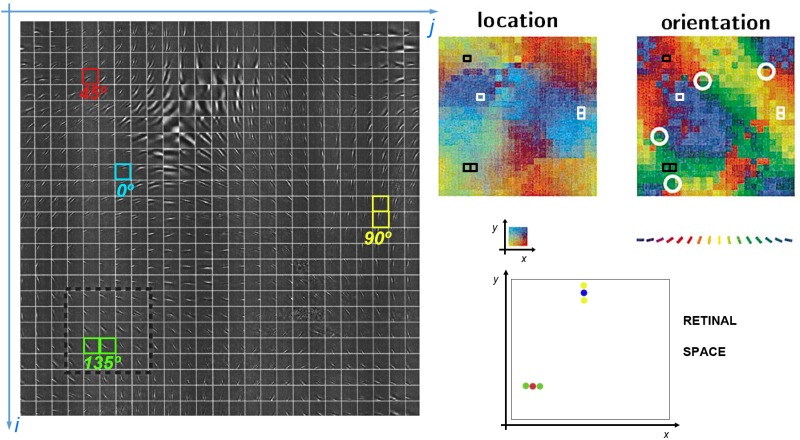
Topology and orientation map in Hyvärinen & Hoyer [28], together with our annotations highlighted with squares. The original source for the left panel (basis vectors in gray scale) and the colored location/orientation diagrams at the top right are Figs 4 and Fig 5b of the paper [28] (reprinted with permission of Elsevier). The highlighted functions with squares and the consequence in the retinal space are the key to question the biological plausibility of the result. The retinal space cartoon in the bottom right illustrates the location (central spatial tuning) of the units in the retina that are highlighted for the discussion (colored squares in the left panel and black and white squares in the diagrams). Here we argue that the interpretation of the blobs in the TICA orientation diagram as *orientation domains* is not possible because, when considering the location of the oriented units back in the retinal space, given two neighboring units tuned to one orientation (e.g. those in green or those in yellow), it is easy to find another unit in between, tuned to an orthogonal orientation (e.g. the one in red or the one in blue). This observation suggests that the TICA orientation domains may be randomly scrambled in the retinal space.

Fig 2 replots the basic result on the orientation domains from TICA in [28] (specifically Fig 4, and part of Fig 5 of the original paper), along with our annotations to discuss the interpretation of the maps. The left panel shows the TICA basis vectors, or processing units, ordered according to the topology that emerges from energy correlations. The top-right panel shows the location and orientation of the receptive fields represented at the left panel. These 2-d location and orientation colored diagrams were built by fitting a Gabor function to each unit in the position (*i*, *j*) in the left panel. Then, each position (*i*, *j*) in the topology (each pixel (*i*, *j*) in the diagram) is depicted using the appropriate color depending on the central location or fundamental orientation of the fitted Gabor function (and the code relating color-location and color-orientation). Finally, at the bottom right part of Fig 2, there is a cartoon of the retinal space to qualitatively illustrate the spatial tuning (or location) of the considered units in the visual field. The spatial distribution of the receptive field centers in this retinal space is the key in the discussion below. The left panel shows that the information theoretic approach by Hyvärinen & Hoyer shares interesting similarities with biological systems: the emerging units have Gabor-like receptive fields (as the V1 simple cells) and the smooth transition in location and frequency along the topology recalls the continuity of the retinotopic iso-orientation domains in the cortex. In fact, the orientation diagram certainly shows locally continuous orientation maps with distinct iso-orientation domains in the topology. The orientation map also displays pinwheels, marked by white circles in the original paper.

However, this interesting behavior may not be automatically applicable to V1 because the correspondence between the TICA topology and the cortical surface is not obvious. Note that the axes of the topology (rows and columns in the left panel, or row/column axes in the orientation diagram at the top-right panel) do not have a smooth spatial meaning as in V1. Note that one finds the same color, i.e. the same retinal location of the receptive field, at distant positions in the *location diagram*, or in the topology. This breaks the global retinotopy that has been described for V1 [4–11]. This result (local but not global retinotopy) was certainly acknowledged as an eventual limitation of TICA in the original paper [28]. However, the implications of this limited retinotopy were not discussed. And no additional analysis has been done to confirm or refute the biological plausibility of the maps emerging in the topology.

Interestingly, a new look at the reported results suggests that the limited retinotopy of the topology may invalidate the biological plausibility of the reported orientation maps: as shown below, this is particularly relevant when considering the spatial distribution of the predicted sensory units in the retinal space (not only in the topology).

The following examples show that it is easy to find strong violations of the continuity of the orientation domains that suggest a random scrambling of the orientation map in the topology when projecting it back to the visual space. If these violations are frequent enough, the locally continuous orientation domains in the topology would disappear in the retinal space (as opposed to the experimental evidence reviewed in Fig 1).

In Fig 2 we highlight two of such cases: consider the sets of sensory units highlighted in the topology using (*a*) green and red squares, on the one hand, and (*b*) yellow and blue squares on the other hand. In the location and orientation diagrams of the top-right panels these units are highlighted using squares in (*a*) black, and (*b*) white, respectively.

In each case (*a* and *b*), the units are quite far in the topology, probably because their responses are almost independent, and they belong to distinct orientation domains. However, they are tuned to a similar spatial location (in the retinal space). The spatial overlapping of the receptive fields can be seen in the left panel, and also because the cells share the same color in the location diagram. Note that in the location diagram *yellow* corresponds to the lower left part of the considered visual field and *light-blue* corresponds to the upper-center region. Actually, the center of the receptive field highlighted in red in the topology could be spatially located *in the visual field* between the two units highlighted in green (e.g. as illustrated in the qualitative retinal space cartoon in the bottom-right). The same happens in the other set of units, the case (*b*): the unit in blue may be surrounded by the two units in yellow. Moreover, the receptive fields of these units, despite being so spatially overlapped, are tuned to nearly orthogonal orientations, as can be seen in the left panel, and also by the very different color in the orientation diagram.

These examples suggest the rupture of the continuity within the orientation domains in the retinal space: (*a*) we have a unit tuned to 45° (in red) overlapped by two units tuned to 135° (in green); and (*b*) a unit tuned to 0° surrounded by two units tuned to 90°. If such mixtures are frequent enough, one foresees a salt-and-pepper distribution of the orientation tuning when plotted in the retinal space. This would not be compatible with the continuity of the orientation maps in the spatial domain reviewed above (Fig 1 and associated discussion in the text). We confirm this guess in the following section.

Qualitatively similar units and orientation maps are obtained using different neighborhood sizes when computing the correlations along the topology [36], or using overcomplete versions of TICA [35]. In both cases [35, 36], the same kind of strong continuity violations highlighted in Fig 2 can be found, so the same questions on the validity of the results arise.

## 2 Results: Topographic ICA does not lead to locally continuous maps in the retinal space

Here we check the distribution of the centers of the receptive fields of the TICA units at the retinal space depending on their orientation tuning, i.e. we study the TICA orientation domains back in the retinal space. This analysis, inspired by the empirical correspondence between a grid in the image space and its counterpart in V1 [8], was not done in [28, 35–37].

Given the straightforward relation between cortical location and spatial tuning in the visual field, Fig 1, a proper theory should lead to locally continuous orientation domains in the retinal space. However, the results in this section show a consistent salt-and-pepper distribution of the oriented TICA units for a variety of visual angles, training sets and sampling frequencies. The whole set of results can be reproduced by using the code and data available at: http://isp.uv.es/code/visioncolor/TICAdomains/TICAdomains.html, and at the public repository https://github.com/neuronavalenciana/OrientationDomainsTICA.

**Fig 3:**
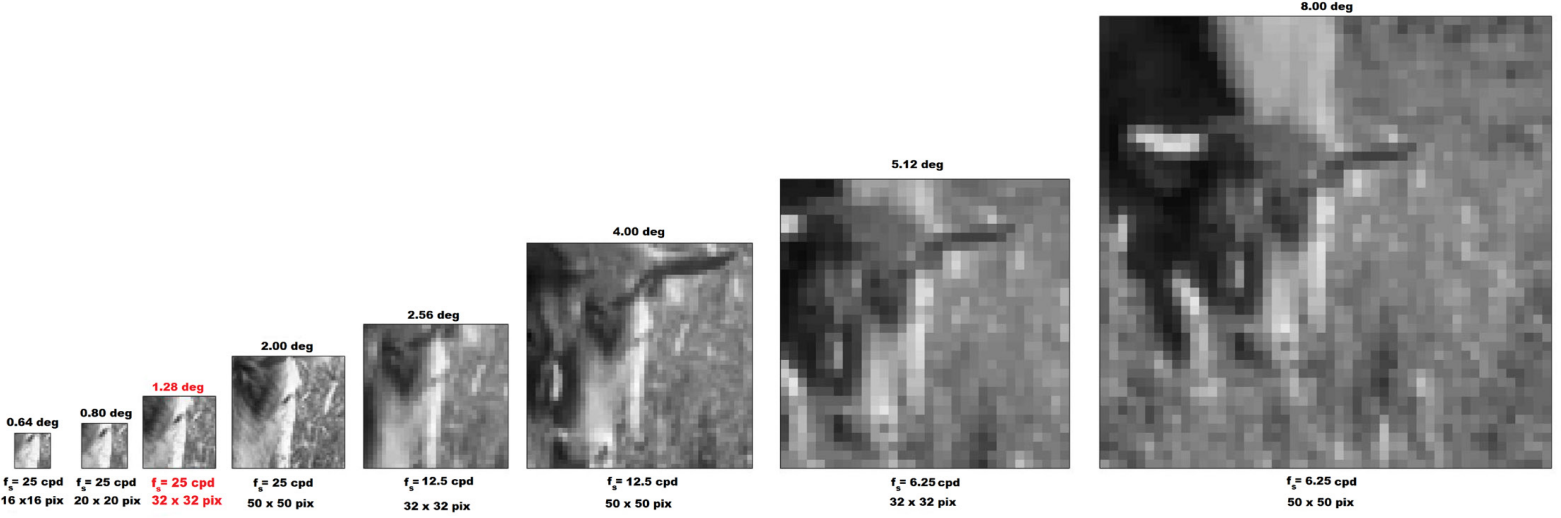
The visual angles considered in our experiments span one order of magnitude: [0.64, 8.00] degrees. In red, the specific resolution considered in [28]. The new experiments largely expand the kind of visual structures considered in a single resolution 32 × 32 set.

In order to check the consistency of the results at different visual scales, we considered different training sets spanning an order of magnitude in visual angle: 0.64, 0.80, 1.28, 2.00, 2.56, 4.00, 5.12, and 8.00 degrees. A wide range of visual angles (and hence a wide range of visual structures) is important in the context of the discussion on local but not global retinotopy in the original paper. See Fig 3 for an example of the kind of structures explored.

In these experiments, all the image samples come from the database in the ImageICA Toolbox [38]. However, since we used different sampling frequencies and block sizes to consider scenes subtending different visual angles, in practice, this means using totally separated training sets. First, we assumed an arbitrary baseline sampling frequency in the database (*f*_*s*_ = 25 cpd). We considered sets of image patches of different discrete sizes with this baseline rate: 16 × 16, 20 × 20, 32 × 32, and 50 × 50; which implies visual angles of 0.64, 0.80, 1.28 and 2.00, degrees. Then, we undersampled the original images using *f*_*s*_ = 12.5 cpd and *f*_*s*_ = 6.25 cpd, and, in both cases, we took discrete sizes of 32 × 32 and 50 × 50; i.e. images with visual angles of 2.56, 4.00, 5.12, and 8.00 degrees. At each of the considered resolutions we gathered 20000 randomly selected patches. In each set we ran the Topographic ICA code in the ImageICA Toolbox [38] with default parameters.

**Fig 4:**
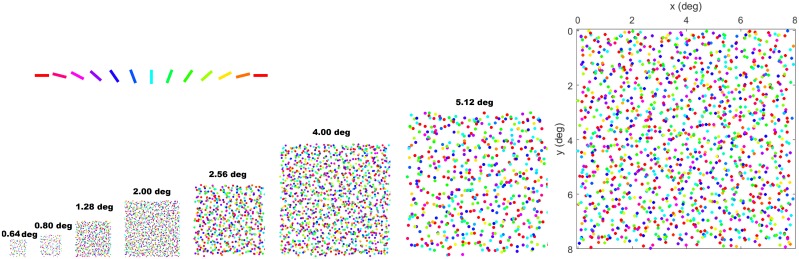
Centers of the receptive fields of TICA units at the retinal space colored depending on their preferred orientation. Results for the different visual angles considered in Fig 3. All the distributions are depicted in the same scale (spanning an order of magnitude in visual angle). Color code for orientation tuning is illustrated by the colored oriented bars at the top. Salt-and-pepper organization is consistently obtained over a range of visual angles, sampling frequencies and training sets.

**Fig 5:**
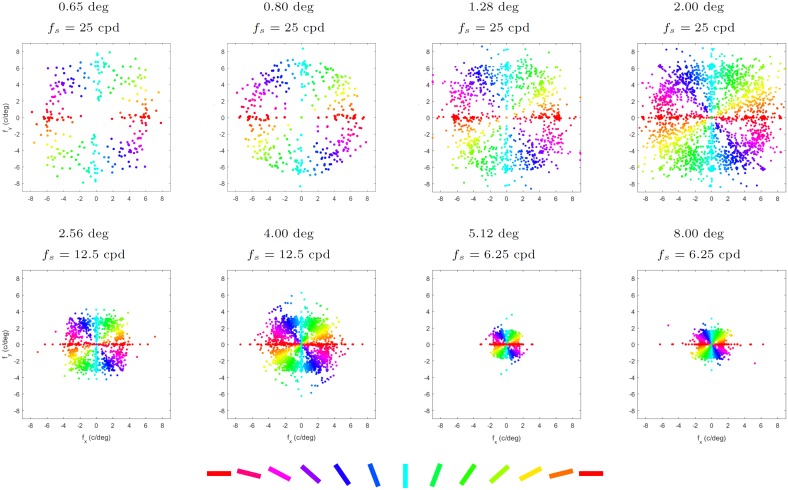
Central frequencies of the TICA units at the Fourier domain colored depending on their orientation tuning for the visual angles considered in Figs 3 and 4. Note that in datasets that have been undersampled the frequency band covered by the mechanisms is smaller as expected by the coarser visual structures due to the antialiasing filter. Color code for orientation is given by the colored bars.

Fig 4 shows the central location of the receptive fields of the TICA units for the different datasets back in the retinal space. Each location is colored according to the orientation preferred by the unit. As an additional check, Fig 5 shows the distribution of the central frequencies of the receptive fields of the TICA units in the Fourier domain. Locations, frequencies and orientations were computed from the parameters of the corresponding Gabor functions fitted to the TICA units. While Fig 5 shows that the TICA units reasonably cover all possible orientations in the frequency range allowed by the considered sampling, Fig 4 confirms the random scrambling intuition anticipated by the observations made about Fig 2 in the Introduction. Qualitatively speaking, the salt-and-pepper-like distributions in Fig 4 do not seem compatible with locally continuous iso-orientation domains in the retinal space: results show a good coverage of the visual field with units tuned to the different orientations, but the units tuned to a similar orientation do not seem to be clustered in locally continuous regions. In the Methods section we present a statistical test that quantitatively rejects the hypothesis of having locally continuous iso-orientation domains in the retinal space.

These salt-and-pepper results are not an artifact of lack of convergence of the iterative algorithm or a poor fit to Gabor functions. In the Methods section we show quantitative and visual illustrations of the proper convergence of the TICA units to Gabor-like functions. The salt-and-pepper distributions do not depend on specific TICA parameter setting either. Free parameters in TICA reduce to [34]: (1) the nonlinearity that is used to enforce the sparsity of the responses during the learning process, and (2) the size of the pooling area after the first stage, which determines the extent of the relations between linear responses. Default settings are [38]: (1) a square-root nonlinearity, and (2) a squared neighborhood of size 5 × 5 for image blocks of size 32 × 32, the dashed square in Fig 2 illustrates this neighborhood. We explored different nonlinearities by varying the exponent in the range [0.2, 0.8], and by considering other saturating functions such as *arc tangent*. We explored the effect of the size of the neighborhood by considering pooling regions of size 7 × 7, 9 × 9, and 11 × 11 for the same image size. For every tested nonlinearity and neighborhood we obtained non-continuous salt-and-pepper distributions of the mechanisms similar to those in Fig 4 (results not shown).

All the above suggests that salt-and-pepper arrangement of edge filters in the retinal space is an intrinsic property of the TICA model when trained on natural images.

In summary, the systematic salt-and-pepper results over a wide range of visual angles, data sets, and algorithm settings, show that the examples of rupture of the continuity of the TICA orientation domains back in the retinal space discussed in Fig 2 are extremely frequent. As a result, this prevents the emergence of locally continuous iso-orientation domains in the retinal space no matter the visual scale. The problem of TICA is that it does not have the degree of retinotopy of the biological systems like the one reviewed in Fig 1.

## 3 Discussion and concluding remarks

### Interaction between similarly tuned neurons has functional advantages

Interaction between neurons tuned to similar features (scale, orientation) in nearby locations has a number of functional advantages, both from a lower-level information transmission perspective or from a higher-level pattern recognition perspective.

From the low-level efficient coding point of view, it is well known that natural scenes display characteristic regularities in linear local-frequency representations: statistical relations between linear responses decrease with the departure in preferred frequency and location in natural images [39–42], and the same happens in natural movies analyzed with spatio-temporal filters, where mutual information decreases with the departure in preferred speed [43]. In fact, the observation of these residual relations after linear ICA was one of the qualitative reasons to propose Topographic-ICA [34]. Interaction between mechanisms tuned to similar features (for instance using divisive normalization [44]) can be thought as a sort of predictive coding to remove these residual redundancies. In fact, the statistical interpretation of this interaction between mechanisms sharing features has been proved useful both to derive biological behavior from efficient image coding [32, 33], and the other way around: to derive efficient image coding from biological behavior [45].

From a higher-level, object recognition perspective, interaction between mechanisms tuned to similar orientations in similar locations have two important advantages. On the one hand, this interaction leads to contrast adaptation [44, 46], and hence, to contrast invariant edge detection which improves recognition performance. On the other hand, detection of large-scale contours requires checking the co-activation of neurona tuned to similar orientation at eventually distant points, therefore, it necessarily requires some sort of selective association between potentially collinear mechanisms [47, 48].

As a side comment, the considered cases of interaction (normalization within iso-orientation domains and association of collinear mechanisms), assume that the interaction takes place between mechanisms that already have a sharp orientation tuning. It is important to stress that the emergence of this sharp tuning may also come from feedback intracortical interactions [49, 50], and not only from the classical feedforward model of Hubel and Wiesel [51] based on the alignment of receptive fields of LGN cells afferent to cortical cells. In this context, approaches based on linear ICA, and also TICA, just assume the classical (and eventually limited) feedforward architecture from the retina [28, 31, 36] leading to a straightforward generation of V1 mechanisms as local linear combination of LGN cells [52]. Therefore, their results do not provide extra insight in the discussion between feedback vs feedforward architectures for the emergence of orientation selectivity.

### Orientation domains simplify interaction between similarly tuned neurons

Interaction between neurona tuned to similar orientations and locations does happen in animals with [53, 54] and without [55] locally continuous orientation maps. The eventual difference is that in the case of having iso-orientation domains, the interaction would occur between close neighbors in the cortex while in the salt-and-pepper situation the interaction pattern is necessarily more complex. This difference has been suggested as a possible explanation of the different complexity of the global connectivity between V1 layers in cats/monkeys versus mice [56].

Moreover, keeping the *mechanisms that should interact* together would probably reduce the wiring requirements when implementing the required interactions (either the divisive normalization for redundancy reduction or contrast invariance, or the association between collinear mechanisms for countour detection). This is consistent with the results in [57] which show that imposing connectivity between mechanisms with very similar tuning, the wiring minimization criterion leads to realistic orientation maps.

### Are TICA maps related to the above functional advantages?

As opposed to other descriptions of the orientation maps which do not take into account the information processing function of the network [16–27, 57], TICA definitely takes it into account since it is based on information maximization and it considers the redundancies between the responses of the first-stage linear mechanisms. Broadly speaking, improved information transmission with regard to linear ICA and improved recognition performance may be functional arguments in favor of the TICA topology. However, the connection between TICA and these specific functional goals is only conceptual.

First, it is not clear that the reported advantages in object recognition when using TICA [58] actually come from the structured organization in the topology. It could also happen that the improved performance is due to the increased invariance of the mechanisms of the second stage in TICA. Unfortunately, the association pattern between the TICA mechanisms (that could be interesting from the biological point of view [47, 48]) was hidden in the machine learning classification stage in [58], so it is difficult to identify the advantage. Moreover, in any case, classification performance is not a part of the learning algorithm in TICA. Second, taking advantage of the eventually ordered structure for further redundancy reduction (e.g. using divisive normalization after TICA) is not a principle included in the TICA learning, so this *simplify-further-processing* argument could not be an explanation for the emergence of the structured maps either.

As a result, despite the functional origin of TICA through infomax, the emergence of structure in the topology in TICA is not directly explained by further redundancy reduction nor by higher-level object recognition arguments.

After all, as suggested by Wilson and Bednar in their excellent review [14], it may also happen that the emergence of the maps does not have a direct functional reason but it is a by-product of other (structural rather than functional) constraints. Nevertheless, despite the interest of the discussion about the usefulness of iso-orientation domains emerging from TICA, the central point of this paper is questioning the biological interpretation of the structure found in the TICA topology, not questioning its functional advantages.

### Conclusion: Orientation maps generated by TICA do not reflect the retinotopy of primate V1 maps

In this work, we saw that a new look at the results reported in [28, 35, 36] suggests a random scrambling of the TICA oriented mechanisms when representing its location in the retinal space. This salt-and-pepper qualitative guess is quantitatively confirmed when systematically checking the organization of the TICA mechanisms in the retinal space (see the Results section). The problem is that TICA does not have the degree of retinotopy (correspondence between location in the topology and location in the retina) observed in the retina-V1 projection of animals with structured orientation maps. As a consequence, the local continuity of the orientation domains in the TICA topology is totally broken back in the image space, so that the hypothesis of existence of a clustered structure there can be reliably rejected (see the Methods section). Therefore, structured orientation maps in the V1 cortex of primates and other mammals cannot be interpreted in terms of topologies generated by TICA.

Given the fact that salt-and-pepper organization in rodents is still a puzzle and a challenge for other mechanistic models, one could think that the ability of TICA to generate this kind of organization could be of some interest to understand those other species. However, the negative result for TICA in primates can not have a positive interpretation for rodents because the TICA retinal organization is salt-and-pepper, but the “cortical organization” in TICA is structured and locally continuous, which is not the case in mice [13].

## 4 Materials and methods

Here we address two methodological issues: (1) the statistical test to show that the set of TICA mechanisms tuned to certain orientation is not clustered in the retinal space as it would be in the case of a structured organization in locally continuous iso-orientation domains; and (2) the appropriate convergence of TICA in our numerical experiments.

### Statistical test of the distributions

The test proposed here is focused on pointing out the uniformity of the set of TICA processing units tuned to certain orientation (the set *S*_*ori*_). Specifically, it addresses the following question: is *S*_*ori*_ similar to a Cartesian grid? If the answer is yes, we are rejecting the presence of clusters in *S*_*ori*_ since a Cartesian grid (*S*_*cart*_) is the less clustered organization one can imagine. In order to use this test in practice, one has to devise a similarity measure and a meaningful reference. As a reference we chose another set lacking of spatial structure: an equivalent finite set drawn from a 2D uniform density function, lets call that *S*_*rand*_. Then, in particular, we check if *S*_*ori*_ is more similar to *S*_*cart*_ than *S*_*rand*_. This question has three possible outcomes: (1) less similar, (2) equally similar, or (3) more similar. If the answer is more or equally similar, *S*_*ori*_ displays no significant clustered structure since its uniformity would be similar or bigger than the uniformity of finite sets drawn from a uniform PDF. In the 1st case we could not extract any conclusion about *S*_*ori*_ spatial organization.

**Fig 6:**
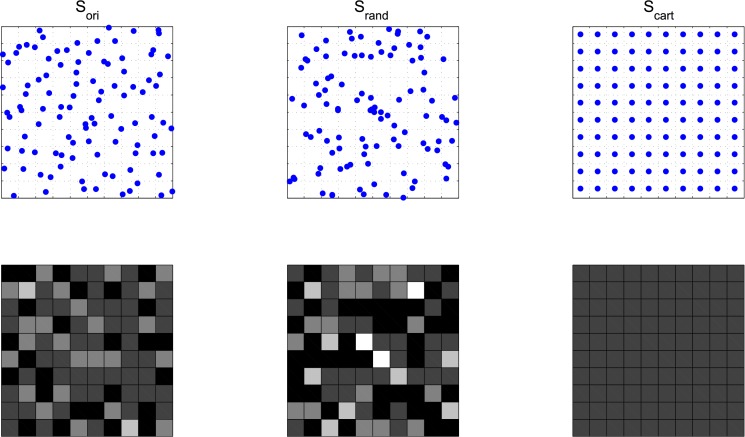
Up left, an example of the distribution of oriented TICA mechanisms in the retinal space, *S*_*ori*_, for certain orientation. Each point corresponds to the center of a mechanism tuned to 135° ± 22°. In this example patch size is 32 × 32, *f*_*s*_ = 12.5, and *K* = 4. Down left is the histogram of the above set. Middle column, an example of a 2-D random sample uniformly distributed, *S*_*rand*_, with the same number of points than *S*_*ori*_, and its corresponding histogram. Right column, an example of Cartesian grid (*S*_*cart*_) and its histogram. The grid of the top figures shows the bins used to built the histograms. In histograms, black corresponds to empty bins while more populated bins are depicted in white.

The proposed test compares the organization of a set of mechanisms (either the considered *S*_*ori*_, or a set of the same size drawn from a 2-D uniform distribution, *S*_*rand*_) against a regular Cartesian grid of the same average density, *S*_*cart*_. Fig 6 shows a realization of these sets, *S*_*ori*_, *S*_*rand*_, and *S*_*cart*_. The similarity measure with *S*_*cart*_ could take into account how many samples of the considered set are close to each node of the Cartesian grid. A convenient dissimilarity measure is the *Kullback Leibler* divergence (*KLD*) between the histograms [59].

The example in Fig 6 shows, that in almost every bin in the histogram *h*(*S*_*ori*_) there is a TICA mechanisms, similarly to *S*_*cart*_. In contrast, *S*_*rand*_ has many empty bins as well many highly populated bins. Note the black and white deviations in the histogram *h*(*S*_*rand*_).

The *KLD* measures how the histograms (*h*(*S*_*ori*_) and *h*(*S*_*rand*_)) depart from the constant value of *h*(*S*_*cart*_). Note that in order to have a lower bound for the divergence, *KLD* = 0, its necessary that the number of samples at the Cartesian grid (and bins of the histograms, *N*_1_ × *N*_2_) its close to the number of samples at the sets *S*_*rand*_/*S*_*ori*_, call it *M*.

Here it is how the test works. Given certain set *S*_*ori*_ of size *M*, we build *N*_1_ × *N*_2_ Cartesian grid that uniformly tiles the retinal plane, where *N*_1_ ⋅ *N*_2_ is the best integer approximation to *M*, $\left(N_{1},N_{2}\in N\right)$. Given the fact that the orientation bandwidth of the mechanisms is in the range [20°, 45°], the literature assumes among 4–6 separate sets of orientated filters. Here we used three different partitions of the continuous [0, 180°] range: *K* = 4, 5, 6, since these correspond to bandwidths of 45°, 36°, 30° respectively. Then, we generate 10^4^ realizations of *S*_*rand*_ of size *M* drawn from a 2-D uniform distribution defined in the same range, and we measure the divergence between the *h*(*S*_*rand*_) of these realizations and *h*(*S*_*cart*_). This allows us to estimate the PDF of the divergence measure, *P*(*KLD*(*S*_*rand*_, *S*_*cart*_)). Finally the divergence *KLD*(*S*_*ori*_, *S*_*cart*_) is calculated and compared with *P*(*KLD*(*S*_*rand*_, *S*_*cart*_)).

**Fig 7:**
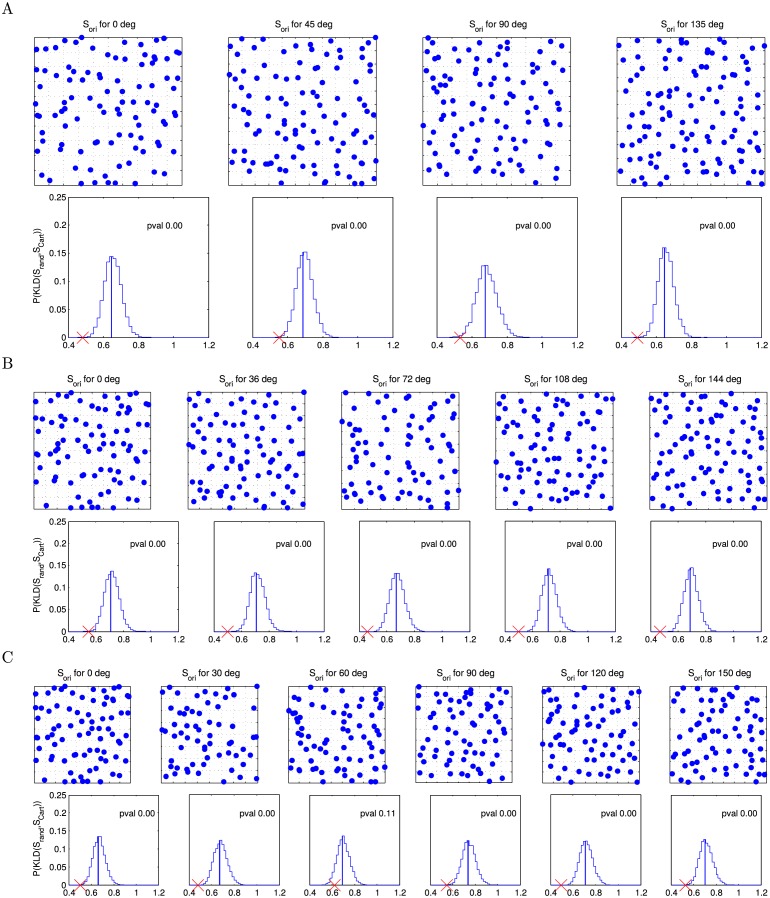
Locations of the TICA sensory units in the retinal space depending on their preferred orientation (sets *S*_*ori*_), and uniformity test results. In order to point out the consistency of the result regardless of the arbitrary partition of the orientation range, here we apply the test splitting the data (the sensory units) according to three different partitions of the [0, 180°] range. We considered different number, *K*, of preferred orientations: *K* = 4 (top panel A), *K* = 5 (middle panel B), and *K* = 6 (bottom panel C). Image data in this example correspond to scenes subtending 1.28 degrees sampled at 25 cpd. Below each set *S*_*ori*_ we represent the corresponding *P*(*KLD*(*S*_*rand*_, *S*_*cart*_)) distribution, a red cross shows the value of (*KLD*(*S*_*ori*_, *S*_*cart*_), and the number displays the corresponding p-value. The blue bars at the distributions show the mean interval of confidence [*μ* ± 2.576 ⋅ *SEM*] (standard error of the mean).

The outcomes of the test are as follows: (1) if *KLD*(*S*_*ori*_, *S*_*cart*_) is significantly bigger than the mean of *P*(*KLD*(*S*_*rand*_, *S*_*cart*_)) no clear conclusion on the structure can be obtained. (2) If *KLD*(*S*_*ori*_, *S*_*cart*_) is similar to the mean of *P*(*KLD*(*S*_*rand*_, *S*_*cart*_)), *S*_*ori*_ would behave similarly as *S*_*rand*_, thus being incompatible with the clustered structure of orientation domains. Finally, (3) if *KLD*(*S*_*ori*_, *S*_*cart*_) falls at the left tail, *S*_*ori*_ is clearly more similar to *S*_*cart*_ than *S*_*rand*_. In this case, the null hypothesis (uniform distribution) can be rejected, since *S*_*ori*_ is actually more uniform than *S*_*rand*_, and even more incompatible with orientation domains. Fig 7 are a representative illustration of the results of the test (similar behavior is obtained in the other cases). In these plots *KLD*(*S*_*ori*_, *S*_*cart*_) (a red cross in the Fig 7) falls below the mean *P*(*KLD*(*S*_*rand*_, *S*_*cart*_)) distribution, so at least we are always in the second case. Even more, in most cases *KLD*(*S*_*ori*_, *S*_*cart*_) falls below the 5% left tail of distribution. This is true regardless of the (arbitrary) number of selected partitions, *K*, patch size and sampling frequency.

This quantitatively confirms the main result (Fig 4): TICA oriented filters display no specific spatial structure in the retinal space, so TICA does not lead to structured orientation maps there.

### Convergence and quality of fit to Gabor-functions

**Fig 8:**
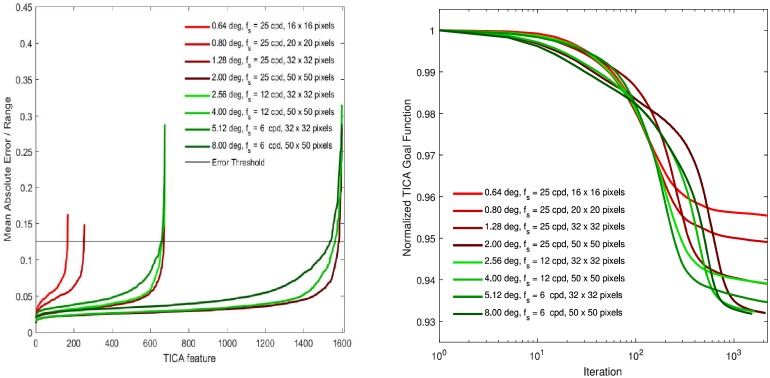
Errors of the fits to Gabor functions (left), and evolution of the TICA goal function (right). In the left plot, features were sorted according to the normalized MAE of the fit to a Gabor function. Different visual angles and resolutions lead to topographies of different size (different number of features). The values of the goal functions during the training of TICA [38] were normalized by the initial value in each case. Curves in red and green correspond to full resolution or to spatially undersampled datasets respectively. Note that normalized MAE is small for most of the features in every dataset. For example more than 95% of the features have a normalized error below 12.5%. Note also that the goal curves in red and green achieve a plateau after 2000 iterations indicating a stable result.

**Fig 9:**
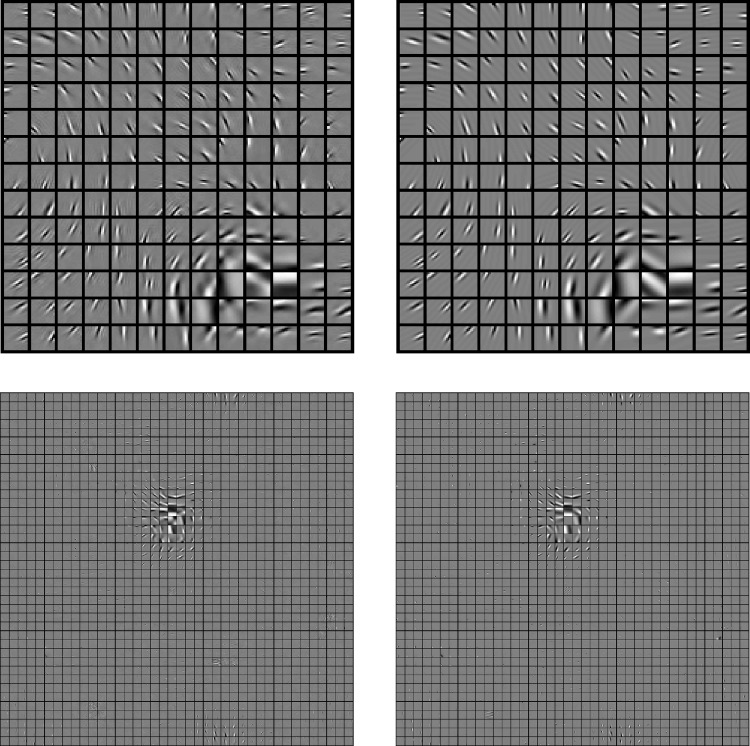
Illustrative results of the TICA basis functions (left) and the corresponding fitted Gabor functions (right) for the training sets corresponding to the visual angles of 0.64 deg (top), and 2 deg (bottom) sampled at *f*_*s*_ = 25 cpd. As can be seen in this example, non-Gabor functions (depicted as flat functions at the right panels) are rare (less than 5%) so fitted parameters are reliable. Qualitatively similar results were obtained for the other datasets.

We achieved satisfactory convergence of the TICA sensory units in 2000 iterations of the algorithm in all the considered training sets. The quality of the convergence and the resemblance of TICA units to Gabor functions is quantitatively and visually illustrated in Figs 8 and 9 respectively. Each (nonparametric) TICA basis function was fitted to a Gabor function, and Fig 8 (left) shows the errors of these fits for all the considered datasets. The departure of the TICA basis function from the corresponding Gabor function is described here by the Mean Absolute Error normalized by the range of values of the TICA receptive field. We refer to this as *normalized Mean Absolute Error* (normalized MAE). Fig 8 (right) shows the evolution of the goal function optimized in TICA [34, 38] in our specific experiments for the different datasets. Appropriate convergence is indicated by two facts: (1) a plateau in the goal function minimized when training TICA, and (2) the emerging receptive fields resemble Gabor functions.

As a convenient reference, the highlighted threshold in the error in Fig 8 (12.5% in normalized MAE), means pretty good agreement with a Gabor function in practice (see the visual examples in Fig 9, which include only functions below this threshold). In all the experiments (main result, Fig 4, and the corresponding uniformity tests) we did not include TICA units with normalized MAE bigger than 12.5%. Most of the TICA receptive fields were well fitted by Gabor functions, less than 5% were not well fitted, so the location, frequency and orientation values in the main result are reliable.
